# A pivotal role for starch in the reconfiguration of ^14^C-partitioning and allocation in *Arabidopsis thaliana* under short-term abiotic stress

**DOI:** 10.1038/s41598-018-27610-y

**Published:** 2018-06-18

**Authors:** Shaoyun Dong, Joshua Zhang, Diane M. Beckles

**Affiliations:** Department of Plant Sciences, University of California, One Shield Avenue, Davis, CA 95616 USA

## Abstract

Plant carbon status is optimized for normal growth but is affected by abiotic stress. Here, we used ^14^C-labeling to provide the first holistic picture of carbon use changes during short-term osmotic, salinity, and cold stress in *Arabidopsis thaliana*. This could inform on the early mechanisms plants use to survive adverse environment, which is important for efficient agricultural production. We found that carbon allocation from source to sinks, and partitioning into major metabolite pools in the source leaf, sink leaves and roots showed both conserved and divergent responses to the stresses examined. Carbohydrates changed under all abiotic stresses applied; plants re-partitioned ^14^C to maintain sugar levels under stress, primarily by reducing ^14^C into the storage compounds in the source leaf, and decreasing ^14^C into the pools used for growth processes in the roots. Salinity and cold increased ^14^C-flux into protein, but as the stress progressed, protein degradation increased to produce amino acids, presumably for osmoprotection. Our work also emphasized that stress regulated the carbon channeled into starch, and its metabolic turnover. These stress-induced changes in starch metabolism and sugar export in the source were partly accompanied by transcriptional alteration in the T6P/SnRK1 regulatory pathway that are normally activated by carbon starvation.

## Introduction

Plants are the primary producers on earth, assimilating carbon dioxide by daytime photosynthesis for the biogenesis of all essential structures. This carbon assimilate is *partitioned* primarily into sugars and starch in the autotrophic ‘sources’^1^ with a portion of the sugars *allocated* to the heterotrophic ‘sinks’ to support growth of the latter^1^. In the absence of photoassimilation, the starch stored in the source is degraded to replenish cellular sugars in order to avoid carbon starvation^2–5^. Therefore, carbon assimilation and utilization is carefully balanced for optimal plant development.

Adverse environmental conditions can disrupt the normal starch and sugars levels with repercussions for the ability of the plant to sustain growth^6^. Drought is associated with reduced starch or sugar levels in source tissues^7–11^. Salinity stress can induce higher starch accumulation in the source or sink of some species^12–17^, but trigger starch reduction in others^18,19^. Similarly, chilling stress is associated with accelerated source-starch accumulation^20–22^ or degradation^23–25^. These observed increases in starch or sugars may be adaptive responses for stress-survival^6^, or may be ‘injury’ responses resulting from the under-utilization of carbon because of growth cessation^26,27^, regardless, documenting these changes is necessary for a deeper understanding of plant stress response.

Feeding plants with ^14^CO_2_ is useful for tracking carbon movement, and can inform on changes in carbon allocation due to stress^17,28–34^. Available data suggests that stress generally accelerates allocation to the sinks as an adaptive response^35^. Salinity increased flux from source to developing fruits in tomato^36^ and to the roots in transgenic rice seedlings^17^. Water-stress elicited a similar distribution pattern in (a) Arabidopsis, with higher ^14^C allocated to the roots^30^, (b) in beans, where ^14^C flux to the pods increased^8^, and (c) in rice, where it stimulated ^14^C mobilization from the stem and allocation to the grain^37^. Additional ^14^C-allocation studies under varied stress conditions could help to clarify whether or not higher source-sink flux is a universal stress response.

The observed changes in local and distant carbon fluxes in plant tissues during stress result from multiple activities – epigenetic, transcriptional, post-transcriptional and posttranslational changes, occurring across different spatial and temporal scales, which must be integrated to deliver a cohesive response to stress^38–42^. The trehalose-6-phosphate/Sucrose non-Fermented Related Kinase 1 (T6P/SnRK1) signaling cascade^40^ may function in this way. It is critical for plant survival under low carbon and energy conditions^43^, in part through changes in starch metabolism^44,45^. The T6P/SnRK1 can also modulate source-sink interactions; therefore, key elements of this regulatory network could potentially be activated for a ‘rewiring’ of whole plant carbohydrate use under stress.

Because of the many issues with respect to plant carbon use under stress that remain unresolved, our aim in this work was to investigate changes in carbon partitioning and allocation in response to short-term drought, salinity, and cold stresses. ^14^CO_2_-labeling of a single source leaf^28^ was used to map whole-plant and intra-tissue changes in carbon use, as it can provide partitioning and allocation data in the same system. Single-leaf labeling permits more accurate tracking of ^14^C-movement than can be obtained by exposing the entire rosette to the label^28^. By comparing plants exposed to different stresses it may be possible to identify convergent and divergent adaptive responses associated with each unfavorable condition. Starch content was also assayed in the source leaf and the roots of the stressed plants and the data were compared to ^14^C-starch fluxes to identify how starch metabolism may be regulated to alter sugar distribution. Finally, the transcriptional activity of key genes in the T6P/SnRK1 pathway was assessed to identify genes associated with changes in carbohydrate levels under abiotic stress. By integrating these data, we present one of the first comprehensive pictures of how Arabidopsis changes carbon flux under short-term environmental stress. This information could be combined with that generated from the wealth of -omics data to broaden our understanding of plant stress response.

## Results

### Time course changes of carbon partitioning and allocation in non-stress treated plants

Our first aim was to investigate how plant source and sink tissues use carbon over the diurnal cycle under normal conditions. One hour before the middle of the day (MD), a single mature, but still developing source leaf was fed with ^14^CO_2_ for 5 min. The labeled source leaf, unlabeled sink leaves, and the roots were harvested separately at MD, at the end of the day (ED), and at the end of the night (EN). MD, ED and EN correspond to 6 h, 12 h and 24 h after dawn. The percentage of ^14^C distributed among the source and the sinks was determined. Within each tissue, the incorporation of ^14^C into the main metabolites pools: sugars, amino acids, organic acids, starch, protein, and ‘remaining insoluble compounds’ (RICs), was established.

**Figure 1:**
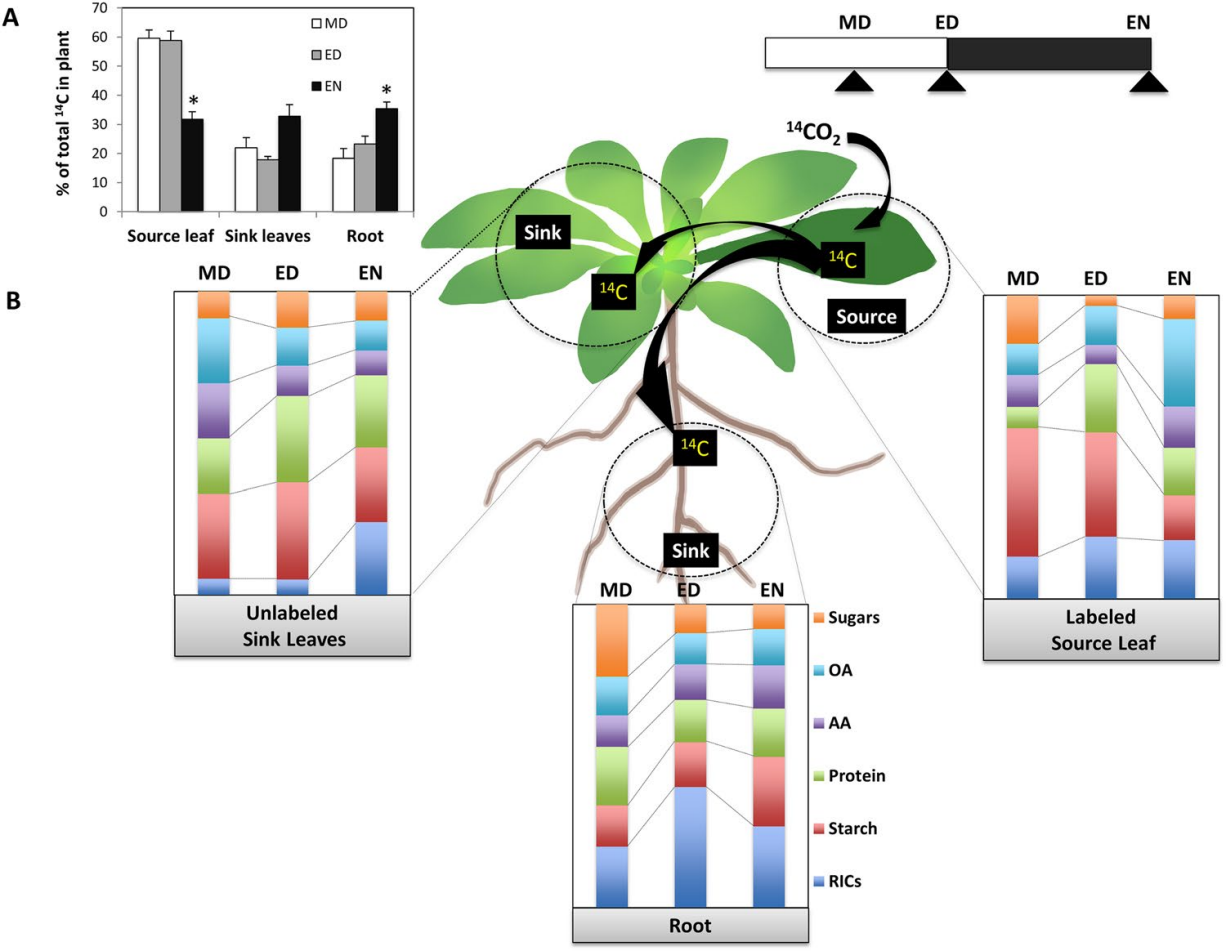
Carbon allocation and partitioning under control conditions. (**A**) Percentage of ^14^C allocated from the labeled source leaf to unlabeled sink leaves and root. The relative amount of ^14^C in each tissue was shown as a percentage of total label in the whole plant. (**B**) Percentage of ^14^C partitioning into metabolite pools. The total ^14^C incorporated into the sugars, starch, amino acids (AA), proteins, organic acids (OA), and remaining insoluble compounds (RICs) in each tissue was set to 100%. MD: midday, ED: end of day, EN: end of night.

First, we calculated the percentage of ^14^C distributed from the source to the sinks. During the day, ~60% of the ^14^C was retained in the source leaf, but by EN, the percentage of total ^14^C was evenly distributed (30%) among all tissues (Fig. 1A). Nighttime export of ^14^C from the source, and its subsequent allocation into the sinks, accounted for the re-distribution.

**Figure 2:**
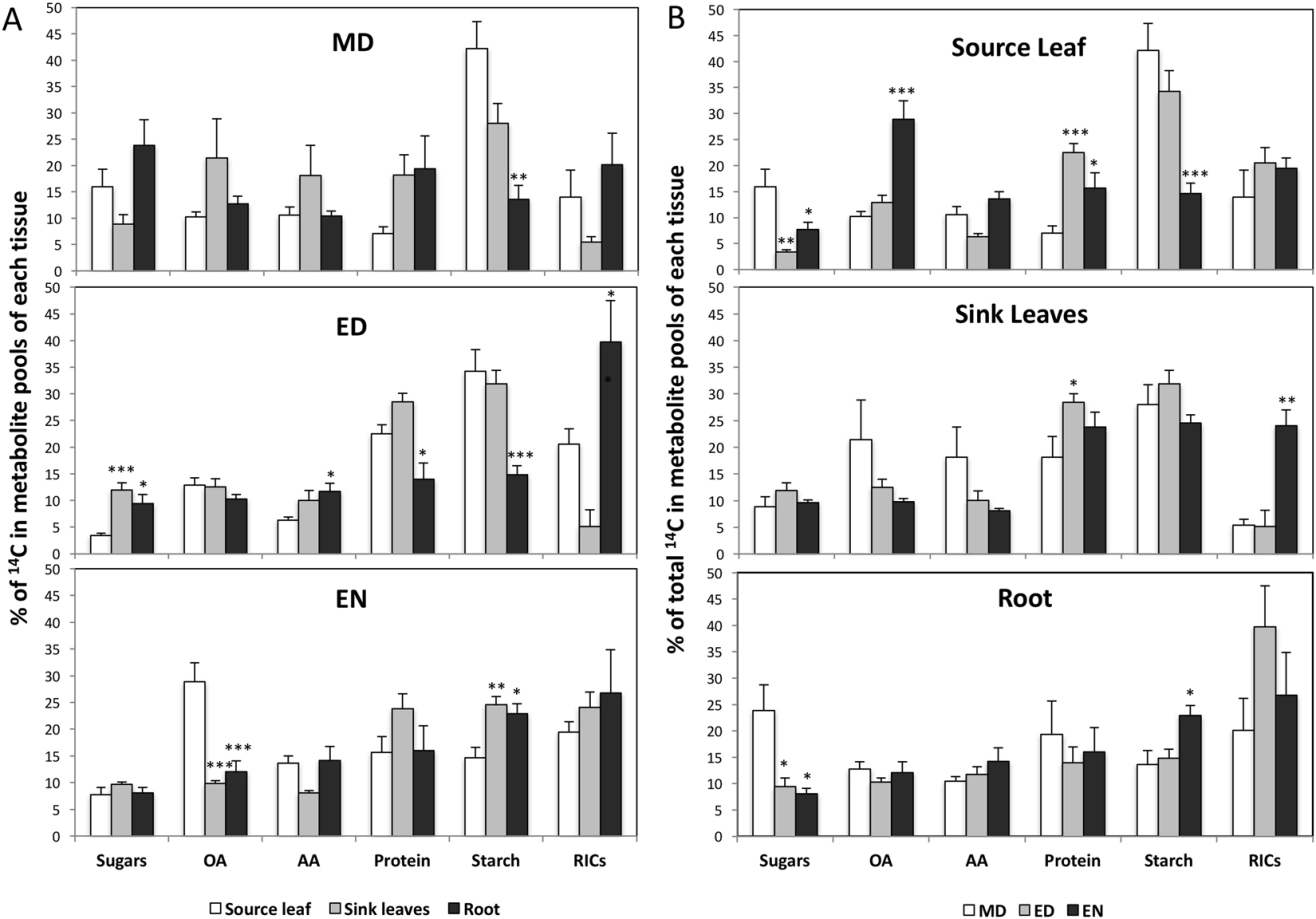
The changes in carbon partitioning over time in different tissues. (**A**). The percentage difference in ^14^C within the metabolite pools among tissues. The asterisks indicate a significant difference between the source leaf and either the sink leaves or root. (**B**) The differential percentage of ^14^C in metabolite pools over time. The asterisks indicate a significant difference between the midday (MD) and end of day (ED) or end of night (EN). The total ^14^C partitioned into sugars, organic acids (OA), amino acids (AA), protein, starch, and remaining insoluble compounds (RICs) in each tissue was set to 100%. (n = 5, ‘*’, 0.01 < *p* < 0.05; ‘**’, 0.001 < *p* < 0.01; ‘***’, 0 < *p* < 0.001).

Second, we examined the ^14^C partitioning between the source and sinks (Figs 1B, 2A) to create a full picture of how allocation and subsequent partitioning were altered. Partitioning in the roots was more dynamic than in the sink leaves, and this difference was amplified most at ED (Fig. 2A). In the roots, there was increased incorporation of ^14^C into metabolites used for growth — i.e. sugars, amino acids, and RICs — and less into those used for storage —i.e. protein and starch — compared to the source. The pattern of ^14^C-partitioning in source leaf vs. roots therefore reflected the prioritization of biological processes in each tissue type. The other change of note occurred at EN, when both sinks incorporated less ^14^C into organic acids but more into starch compared to the source. This may indicate that the sinks had greater sufficiency with respect to carbon (due to sugar import) with a relatively reduced need for organic acids as sources of energy compared to the source.

Finally, we examined changes in ^14^C-partitioning over the diurnal cycle (Fig. 2B). Data at ED and EN were compared to that generated at MD to fully assess how the day-night cycle affected carbon partitioning in different tissues. The metabolic pools in the source leaf were variable, while those in the sinks were relatively stable. Relative to MD, there was less ^14^C in the sugar and starch fraction, but an almost 2-fold greater flux into organic acids at EN in the source. Organic acids may serve as the primary substrate for respiration after reductions in the sugar pool^1^. In the roots, at EN, the ^14^C percentage in sugars decreased, but increased in starch. This indicates that the starch in the roots was accumulated constantly during the diurnal cycle, with more accretion during the night than the day. In contrast, in the sink leaves, the carbon flow into sugars and starch were stable at EN, but there was a 4-fold increase in the ^14^C partitioned into the RICs, suggestive of nighttime growth processes.

### Carbon partitioning and allocation under abiotic stress

How stress altered Arabidopsis carbon use over the diurnal cycle at the cellular and whole plant level was examined. Arabidopsis seedlings were exposed to salinity stress using 100 and 200 mM NaCl, to osmotic stress using 150 and 300 mM mannitol, and to cold stress by exposing roots to 0 °C cold at the beginning of photoperiod. After 5 hours of stress treatment, a single mature source leaf was fed with ^14^CO_2_ for 5 min. Sampling was done as previously described.

#### Osmotic stress

Carbon allocation was negatively affected by osmotic stress, and the inhibition grew in severity as the stress progressed (see Supplementary Fig. S1). By EN, mild (150 mM) and severe (300 mM) mannitol stress increased the percentage of ^14^C in the source, and decreased it in the roots (see Supplementary Fig. S1). This could reflect reduced carbon export due to enhanced source activities, inhibited carbon export from the source, reduced sink strength, or a combination thereof under osmotic stress.

**Figure 3:**
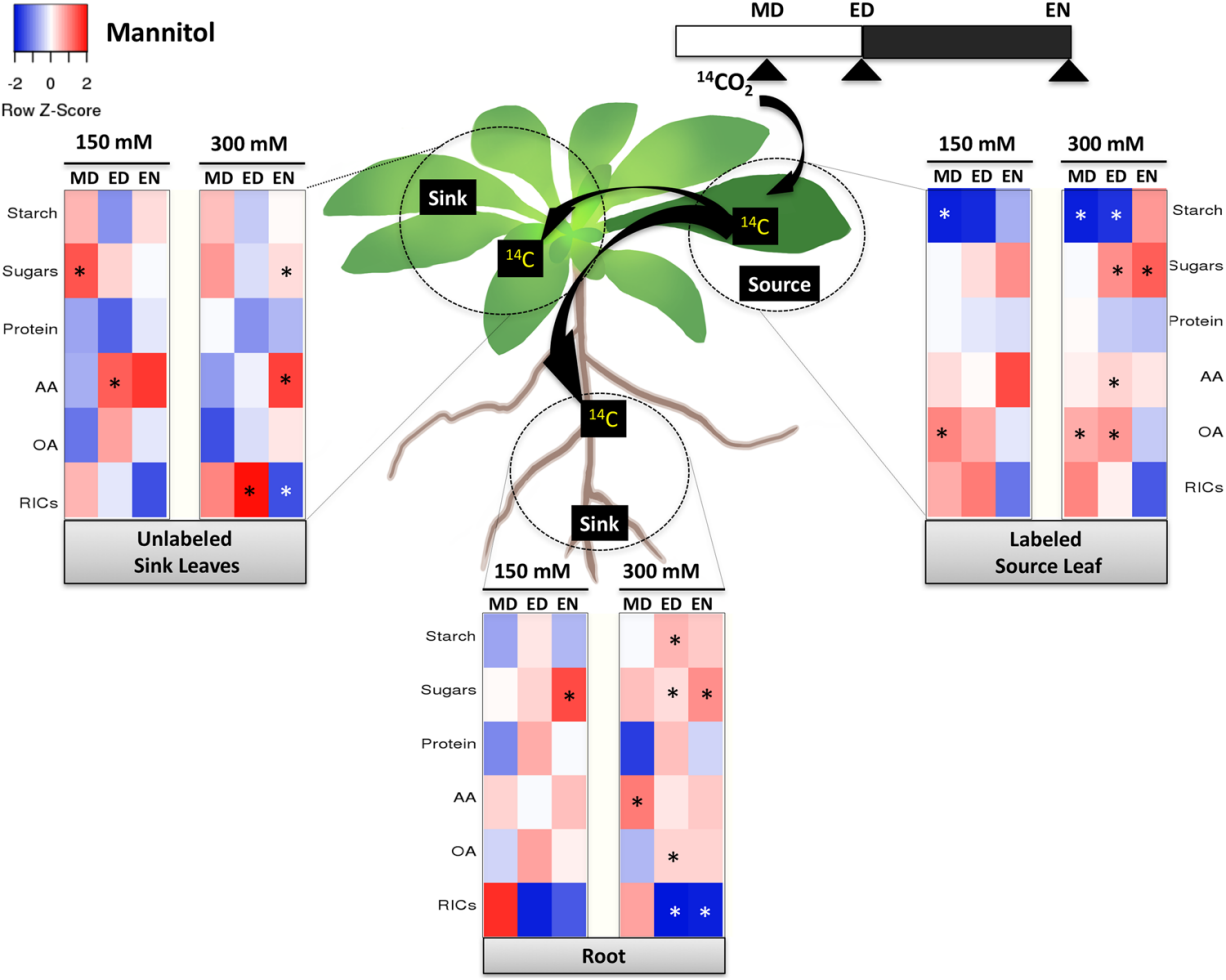
A diagram showing changes in ^14^C partitioning within each tissue under osmotic stress. Heat map showing the difference in ^14^C partitioning over time between the stress-treated samples and control. The incorporation of ^14^C into sugars, starch, amino acids (AA), protein, organic acids (OA), and remaining insoluble compounds (RICs) pools in each tissue was set to 100%. The difference in ^14^C percentage between mannitol-treated (150 or 300 mM) and control in each metabolite was calculated, and the differences were shown as a heat map. Red represents a higher percentage than the control, while blue represents a lower percentage than the control. The asterisk indicates a significant difference between the control and mannitol-treated plants (n = 5, *p* < 0.05). MD: midday, ED: end of day, EN: end of night.

Carbon partitioning within the source was also modulated to a greater extent than in the sinks (Fig. 3). At MD, both mild and severe osmotic stress reduced the ^14^C-partitioned into starch but increased ^14^C-partitioning into organic acids in the source, presumably for respiratory use. Six hours later, only severe osmotic stress had this effect leading to greater ^14^C flux into osmoprotectants — sugars, organic acids, and amino acids — at the expense of the storage compounds (i.e. starch). The ^14^C-flux into these osmoprotectants also increased in both sinks at the expense of the RICs, with the latter decreasing drastically (by 34.7% at ED and 14.3% at EN) in the roots.

#### Salinity stress

**Figure 4:**
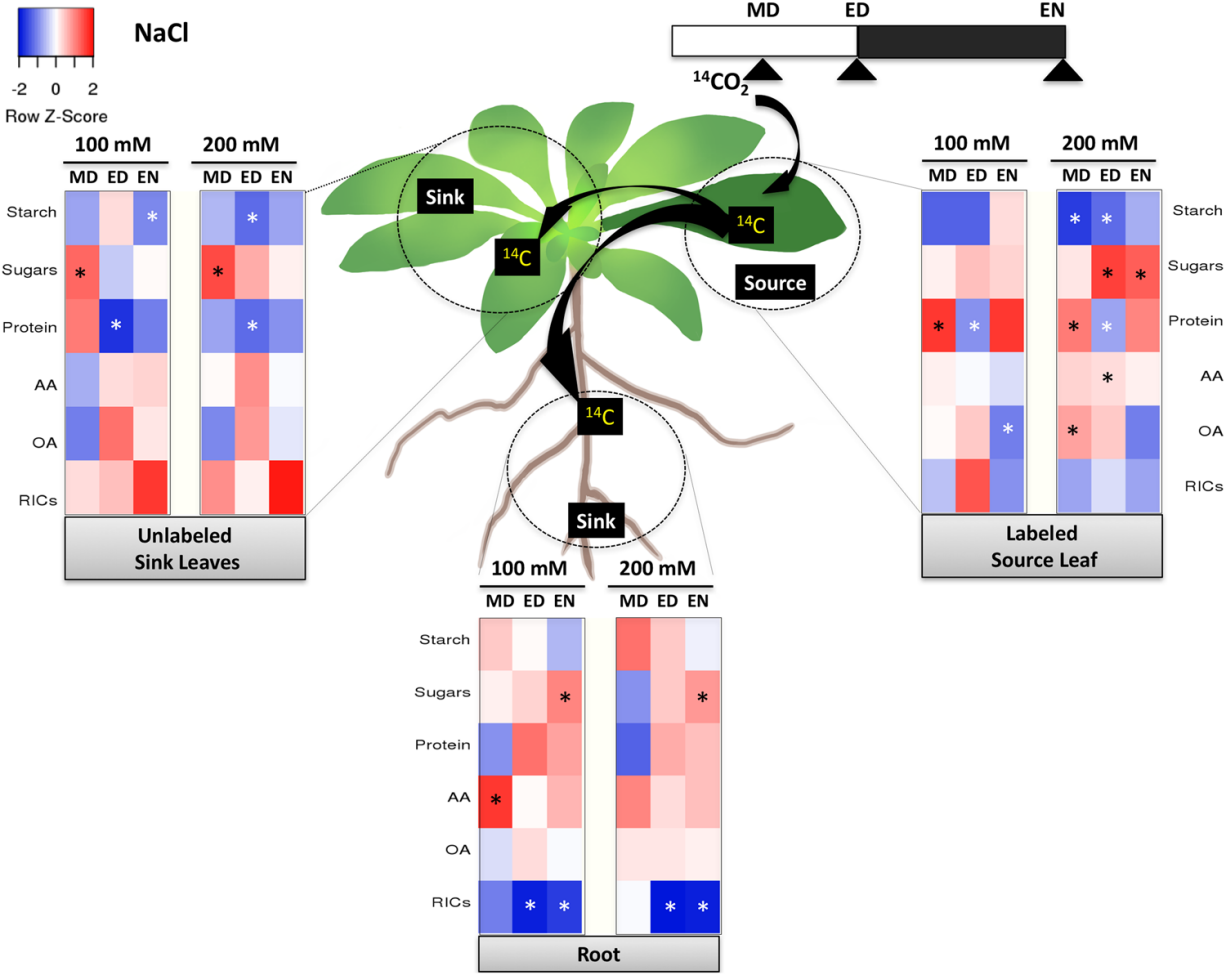
A diagram showing changes in ^14^C partitioning within each tissue under salinity stress. Heat map showing the difference in ^14^C partitioning over time between the stress-treated samples and control. The incorporation of ^14^C into sugars, starch, amino acids (AA), protein, organic acids (OA), and remaining insoluble compounds (RICs) pools in each tissue was set to 100%. The difference in ^14^C percentage between NaCl-treated (100 or 200 mM) and control in each metabolite was calculated, and the differences were shown as a heat map. Red represents a higher percentage than the control, while blue represents a lower percentage than the control. The asterisk indicates a significant difference between the control and NaCl-treated plants (n = 5, *p* < 0.05). MD: midday, ED: end of day, EN: end of night.

The most obvious change was the percentage of ^14^C allocated from source leaf into roots, which decreased significantly by EN under both mild (100 mM) and severe (200 mM) NaCl stress (see Supplementary Fig. S2). The ^14^C-use in source leaf was more responsive to salinity compared to the sinks (Fig. 4). Severe salinity stress decreased ^14^C-partitioning into starch but increased partitioning into sugars, amino acids, and organic acids during the day in the source. At MD, more ^14^C was partitioned into sugars in the sink leaves, but 6 h later at ED the ^14^C in sugars was stable, with reduced flux into starch and proteins. This indicates that 12 h after the stress treatment, carbon was diverted from storage and preferentially partitioned into sugars for osmoprotection. In the roots, less ^14^C was partitioned into the RICs at ED and EN compared to the control, which suggest a shift away from investing ^14^C into resources normally used for root growth under salinity. This may have led to increased ^14^C accumulation into sugars at the end of night because they were under-metabolized. Interestingly, proteins were the only metabolite affected by both mild and severe salinity stress in both source and sink leaves, while it was unchanged in the roots. Carbon flux into this pool decreased compared to the control at ED in both source and sink leaves. Further, unlike sink leaves, the source had increased ^14^C label in protein at MD (Fig. 4).

The changes in ^14^C partitioning and allocation in response to different levels of salinity stress are summarized as follows: (a) the source leaf partitioned less ^14^C into storage compounds (starch, or protein) but more ^14^C into osmoprotectants (sugars, amino acids, organic acids) in response to severe salinity stress; (b) sink tissues showed a differential response to salinity stress: similar to the source leaf, the sink leaves showed reduced ^14^C in storage compounds, however, roots tissue had reduced ^14^C in structural compounds; and (c) the amount of ^14^C imported into roots tissue was inhibited by salinity; this might be due to reduced sink activity, inhibited phloem transport, or a combination thereof.

#### Cold stress

**Figure 5:**
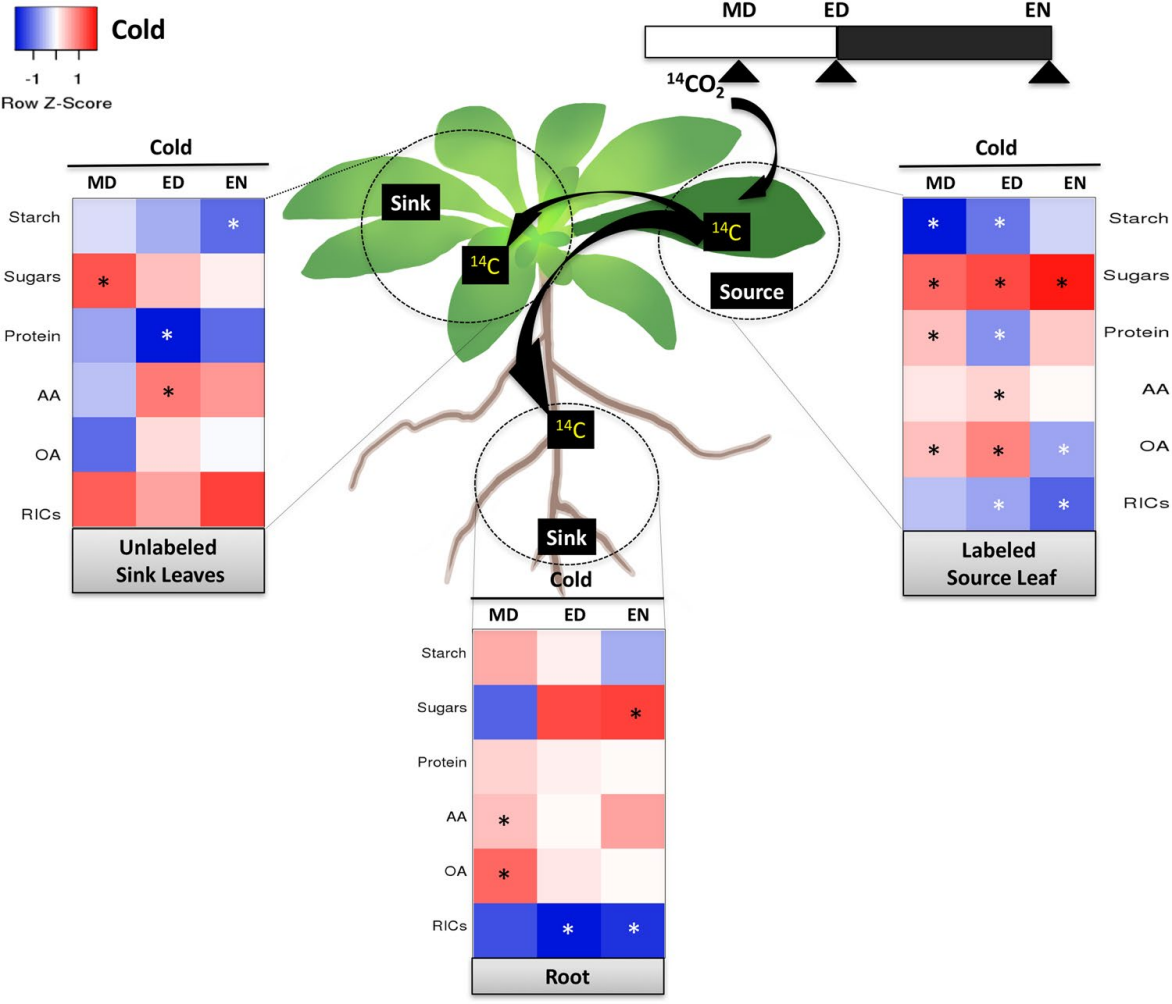
A diagram showing changes in ^14^C partitioning within each tissue under cold stress. Heat map showing the difference in ^14^C partitioning over time between the stress-treated samples and control. The incorporation of ^14^C into sugars, starch, amino acids (AA), protein, organic acids (OA), and remaining insoluble compounds (RICs) pools in each tissue was set to 100%. The difference in ^14^C percentage between cold-treated and control in each metabolite was calculated, and the differences were shown as a heat map. Red represents a higher percentage than the control, while blue represents a lower percentage than the control. The asterisk indicates a significant difference between the control and cold-treated plants (n = 5, *p* < 0.05). MD: midday, ED: end of day, EN: end of night.

The percentage of ^14^C in root tissues was significantly reduced by cold stress at the end of night, showing similarity to tissues under osmotic and salinity stress (see Supplementary Fig. S3). Carbon allocation was not affected by low temperature during the day (see Supplementary Fig. S3), but carbon partitioning was highly regulated in the source leaf (Fig. 5), especially at the end of day. The most notable difference was that the ^14^C-flux into starch and RICs decreased relative to the control plants. The decrease in starch was high at MD but lessened during the diurnal cycle, while the opposite was true for the RICs, where inhibition intensified over the day. In the source, there were also higher fluxes into sugars, amino acids, and organic acids from MD to ED. Cold also triggered increased ^14^C into the protein pool at MD, and decreased it at ED. At EN, the ^14^C in RICs strongly decreased, with a corresponding strong increase in sugars. Cold stress therefore stimulated more ^14^C partitioning into sugars over the diurnal cycle in the source leaf.

The sinks were less affected by cold than the source. In sink leaves, there was increased carbon flow into sugars during the day and decreased carbon into starch at night, with no difference in RICs. In contrast, the roots had increased ^14^C in the sugar pool at night, and reduced partitioning into the RICs (at ED by 20.4% and at EN by 11.6%). This change of ^14^C partitioning suggests reprioritization of reserves with a greater flux towards sugars for osmoprotection at the expense of other pathways.

### Steady-state accumulation of starch vs. partitioning under abiotic stress

The ^14^CO_2_ labeling experiment showed that starch is the most dynamic metabolite pool that changed under all types of abiotic stresses used in this study. ^14^C-flux into starch was down-regulated by abiotic stress, and the regulation depended on the time of day and tissue type examined.

Under control conditions, ^14^C-partitioning into starch was stable during the day but decreased at night in the source leaf (see Supplementary Fig. 7A). However, this pattern was disrupted under salinity and cold stress due to reduced carbon flow into starch. In contrast to the source leaf, ^14^C in starch in sink leaves did not change during the day even under stress. In roots, the percentage of ^14^C into starch normally increased by EN, and interestingly, this partitioning was maintained under osmotic stress, but not under salinity and cold stress. Source and sink tissues therefore partitioned carbon into starch differently in response to abiotic stress (see Supplementary Fig. 7B). Source leaf showed reduced ^14^C partitioning into starch at MD under both mild and severe salinity and osmotic stress, and at ED under severe stress only. Sink tissues had very little changes of ^14^C in starch under stress.

**Figure 6:**
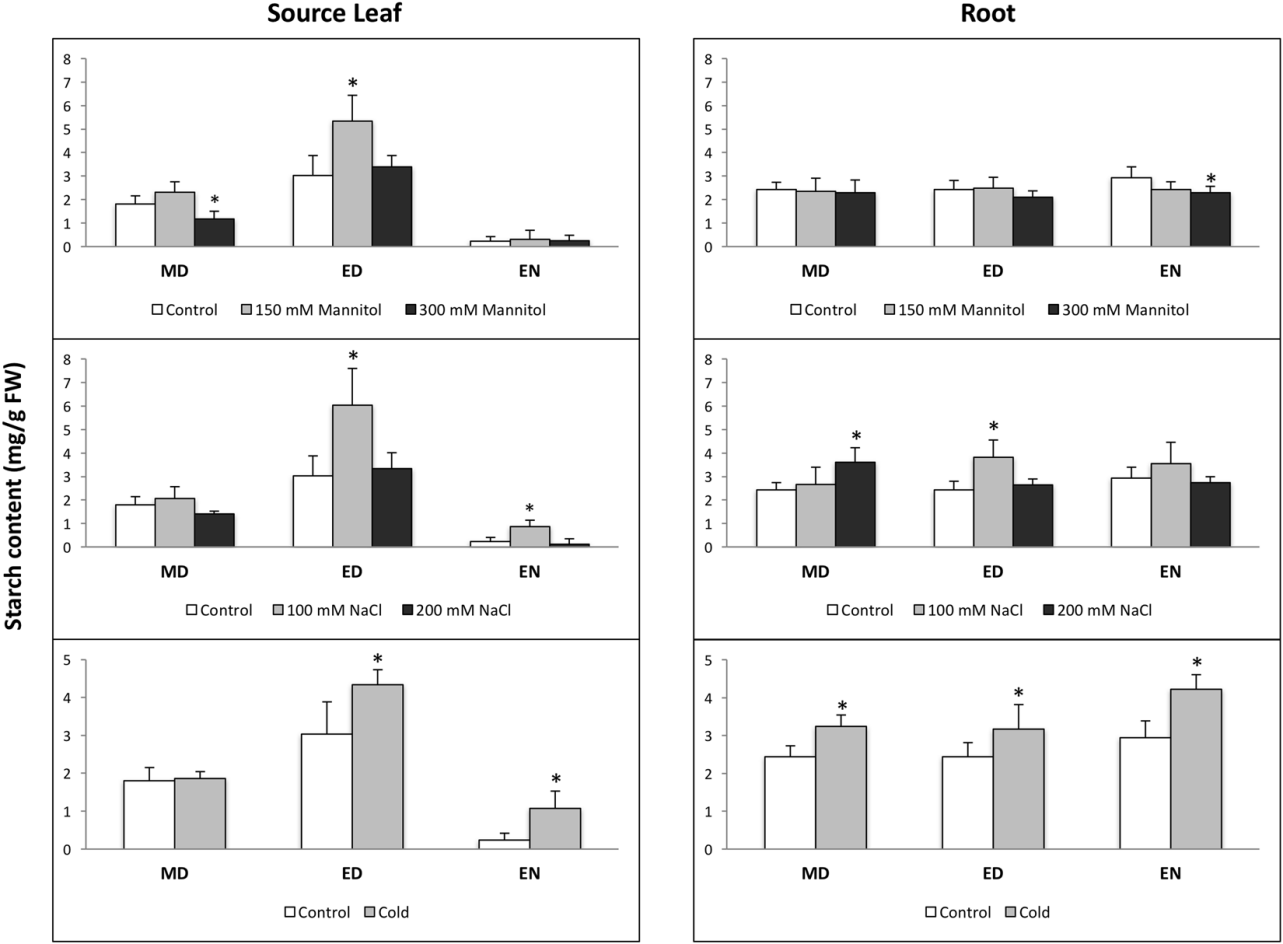
Starch contents in source leaf and root under stress. Starch was assayed in plants exposed to mannitol (150 or 300 mM), NaCl (100 or 200 mM), and cold stress for 6 h (MD: midday), 12 h (end of day), or 24 h (end of night). The asterisks indicate a significant difference between the stress-treated and control samples. MD: midday, ED: end of day, EN: end of night (n = 5, *p* < 0.05).

Since the plants were labeled 5 h after the stress treatment, the ^14^C flux into starch cannot provide a whole picture of starch metabolism changes during the entire stress period. It only informs on percentage change in partitioning and allocation. Therefore, absolute starch content measurements were made in source leaf and roots (Fig. 6), to determine changes in accumulation over the time-course. Comparison of the variations in the percentage of ^14^C partitioned into starch with changes in starch accumulation could indicate if there are additional regulatory mechanisms leading to turnover, i.e. simultaneous synthesis and degradation.

Cold, mild osmotic and salinity stress triggered enhanced starch accumulation at ED in the source. Twelve hours later (EN), only cold and mild salinity kept starch accumulation high relative to the non-stressed control. In comparison, the ^14^C that partitioned into starch decreased from ED to EN (see Supplementary Fig. 7A), thus, the increased starch content observed might be due to inhibited starch degradation early in the day. A similar pattern was found in the roots — higher starch accumulation even though there was no change in the percentage of ^14^C partitioned to starch over the same period. Therefore stress may have reduced the rate of starch turnover in the roots.

### Transcript level of genes integrate carbohydrate and abiotic stress

Because the starch-sugar interconversion in source leaf was acutely regulated in response to environmental cues, we further examined if changes in starch metabolism and sugar export was accompanied by the regulation of the known T6P/SnRK1 stress signaling pathway genes. The transcript level of five selected genes in source leaf exposed to 300 mM mannitol and 200 mM NaCl stress, which triggered the most dynamic changes in starch metabolism, were evaluated. These genes are involved in starch synthesis, sucrose transport, and are components of the T6P/SnRK1 stress signaling pathway. *AtTPS1* encodes trehalose-6-phosphate (T6P) synthase^46^, *AtSnRK1.1* and *AtSnRK1.1* encode two major isoforms of SnRK1^39–42^, the central players of the T6P/SnRK1 pathway. *AtAPL3* encodes the large subunit of ADP-glucose pyrophosphorylase (AGPase) that catalyzes the first committed step in starch biosynthesis. *AtSWEET11* encodes a transporter that exports sucrose from leaf mesophyll cells into the phloem for transport to sinks^47–49^.

**Figure 7:**
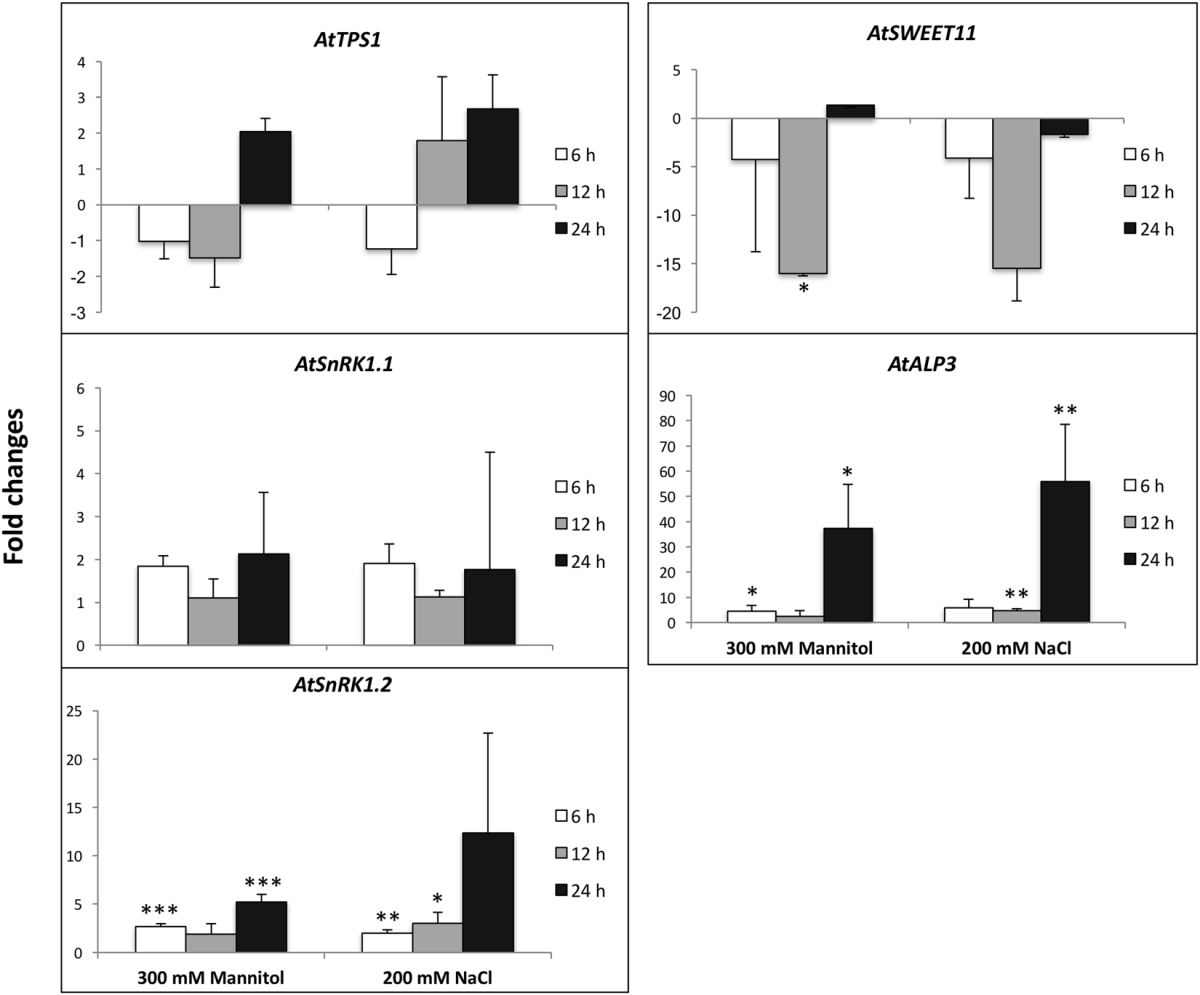
Quantification of transcripts from the *AtTPS1*, *AtSnRK1.1*, *AtSnRK1.2*, *AtSWEET11*, and *AtAPL3* genes. The transcript levels of genes in non-stress treated control were set as 1. The vertical axis indicates the relative amount of mRNA of each gene in stress-treated samples compared with that in non-stress treated control. RNA samples were extracted from plants treated with 300 mM Mannitol or 200 mM NaCl for 6 h, 12 h, or 24 h. Average qPCR data were derived from nine data measurements for each sample. Error bars represent the standard deviation. The asterisks indicate statistically significant difference of transcripts level between genes in the control and stress-treated plants (‘*’, 0.01 < *p* < 0.05; ‘**’, 0.001 < *p* < 0.01; ‘***’, 0 < *p* < 0.001).

Our measurements showed that *AtSnRK1.2* was up-regulated by osmotic and salinity stress after 6 hours of treatment (Fig. 7). However, the transcription of *AtTPS1* and *AtSnRK1.1* did not change. *AtSWEET11* was down regulated by severe osmotic stress at the end of day. *AtAPL3* was up-regulated at MD and at EN by 300 mM mannitol stress, and was up-regulated from ED to EN by 200 mM NaCl.

## Discussion

### Carbon allocation and partitioning is regulated by abiotic stress

Our overall aim was to develop a comprehensive map of time-dependent changes in carbon allocation and partitioning, to see how these processes were affected under different stresses.

In our study, the ^14^C partitioning in source and different types of sinks (leaves, and roots) over the diurnal cycle was examined. Under control conditions, ^14^C distribution into different metabolic pools in source and sink tissue, followed expectation based on previous knowledge^29^. Source leaf, sink leaves and roots tissues showed different carbon partitioning, with most dynamism in the source. Most carbon in source leaf flowed into storage compounds (starch), and less flowed into structural compounds (e.g. cell walls) during the day. This result is similar to a previous study^29^. The roots also generally incorporated more carbon into RICs while sink leaves partitioned more into starch. This indicates a clear differentiation in carbon use between sink leaves and roots.

Carbon allocation to the sinks was modulated by all abiotic stress conditions used in our study (see Supplementary Figs S1–S3). Stress conditions should reduce photosynthetic capacity and carbon available for export. Knolling *et al*. showed that carbon export from the source to sink leaves was reduced in Arabidopsis experiencing dark-induced carbon-starvation^29^. Our study included roots, which is a stronger sink than leaves. We found that the C-fluxes into the roots were more vulnerable to stresses than those into sink leaves (see Supplementary Figs S1–S3). Furthermore, plants might regulate carbon allocation differently in response to long-term and short-term stresses requiring caution when making comparisons between studies. Durand *et al*.^30^ observed a higher percentage of ^14^C allocated into roots in the long-term water deficit stressed Arabidopsis. However, data from plants exposed to short-term stress (24 h) in our study and plants exposed to a 16 h night^29^ showed the opposite results: reduced percentage of ^14^C exported into roots. This underscores that timing, intensity, and type of stress regulate carbon allocation differently, even if some stresses show similar responses.

**Figure 8:**
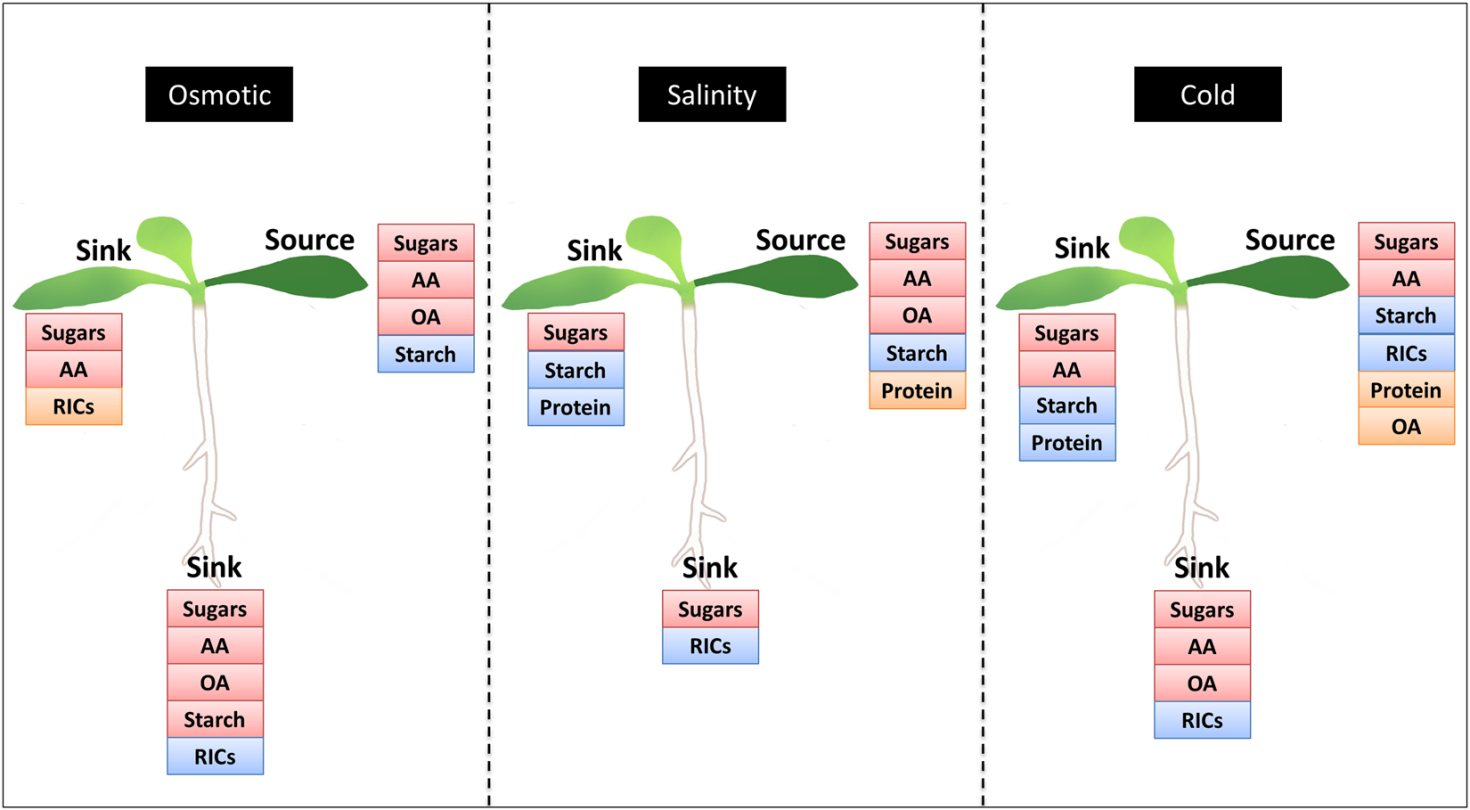
A model of the common and specific responses of stress-triggered alterations in metabolite pools. Changes in ^14^C partitioning into metabolites by cold (0 °C), 300 mM mannitol, and 200 mM NaCl were compared over the diurnal period. Colored boxes indicate the percentage change in ^14^C flow under stress: metabolites in red boxes increased; in blue boxes decreased and metabolites within orange box increased and then decreased (*p* < 0.05) over the 24 h period.

Osmotic, salinity, and cold stress all triggered complex changes in carbon partitioning and shared some commonalities (Fig. 8). All stresses increased the carbon partitioned into sugars in both source and sink tissues. They also decreased the ^14^C partitioning into starch in the source leaf while increasing organic acids and amino acids. Each stress had a more dramatic impact on source leaf than the sink tissues, with most changes occurring within the first 12 h of stress application. The abiotic stresses used here all triggered decreased ^14^C flux into RICs (the carbon pool representative of growth) in the roots (Figs 3–5). Among the major metabolites pools affected, changes of carbohydrates were most consistent. Kolling *et al*.^29^ observed an increase of ^14^C into sugars and a reduction of ^14^C flux into the RICs pool in both source and sink leaves. However, in our study, the increased ^14^C flux into sugars in the source leaf was due to the re-partitioning of ^14^C from storage compound (i.e. starch), while the increase in the sink could be explained by the reduced ^14^C partitioning into structural compounds (i.e. cell walls).

Different abiotic stresses may uniquely regulate carbon use (Fig. 8). Only cold stress caused a decrease in ^14^C in RICs in source leaf. Osmotic and cold stress, but not salinity stress, increased ^14^C flux into organic acids and amino acids in root tissues, and enhanced ^14^C into amino acids in sink leaves. Only cold and salinity stress, provoked changes in ^14^C in protein in source and sink leaves. Higher ^14^C in protein at the early stage of the stress progression may be due to the accumulation of stress-responsive proteins and enzymes. When stress continued, storage compound like the storage, cytosolic, and vacuolar proteins are degraded and recycled to provide energy and substrates for respiration^50–55^.

### The regulation of starch metabolism in response to abiotic stress

The regulation of starch accu-mulation by abiotic stress in Arabidopsis were mainly studied during the day and only focused on leaves. Mild-to-moderate mannitol stress (25 to 200 mM) triggered starch accumulation^56–58^, whereas higher mannitol concentrations (300 or 450 mM) or severe drought led to decreased leaf starch^32,59–61^. Moderate-to-severe salinity (150 mM NaCl) decreased starch in Arabidopsis leaves^62^. Cold stress induced starch accumulation in leaves in some studies^20–22^, while decreased starch accumulation in others^23^. Our study differentiated between source and sink tissues, and starch content was regulated by abiotic stress in both. There was a lack of congruency in the starch accumulation and ^14^C-starch partitioning under cold, mild osmotic, and salt stress in source and roots (Fig. 6 and Supplementary Fig. 7). Higher starch content in sink under stress might be due to decreased starch utilization. In the roots, more ^14^C accumulated as sugars because of the decreased ^14^C partitioning into structural compounds. In this case, it might not be necessary to degrade starch into sugars.

Starch, as a sugar reservoir, regulates plant carbon balance to avoid potential famine^5,6,63–65^. Maintaining sugar levels by cycles of synthesis and degradation of starch could permit metabolic flexibility with respect to starch-sugar interconversion. The sugars so produced may act as Reactive Oxygen Species scavengers, osmoprotectants and be an immediate source of carbon and energy to mitigate against stress^66–70^. Sugar conversion to starch in leaves may prevent feedback inhibition of photosynthesis, and higher starch in the roots could help gravitational response under stress, and enhance biomass for better foraging^17,38^.

### Transcript level of genes integrating carbohydrate and abiotic stress

Transcripts levels of T6P/SnRK1 pathway genes were regulated by abiotic stress in this study. *AtSWEET11*, one of the sucrose transporters, is important in whole-plant carbon allocation^30,47,71^. It is expressed when sucrose export is high^30^ and repressed during osmotic stress in Arabidopsis leaves, when presumably export is lower^71^. In our study, *AtSWEET11* was down regulated by osmotic stress at the end of day, which suggests that the export of sugar to the sinks was inhibited. The repression was likely due to feedback inhibition by excess sugars, this is supported by our data, which showed more ^14^C in sugars in the source leaf at ED, and decreased ^14^C imported into roots (see Supplementary Fig. S2). *AtAPL3* was shown to be up-regulated by 150 mM NaCl stress in Arabidopsis^62^. Our study also observed the up-regulation of *AtAPL3* by 200 mM NaCl, and 300 mM mannitol stress. Interestingly, the percentage of ^14^C partitioned into starch was reduced, and the end point starch content remained unchanged. Changes in the post-transcriptional regulation of AGPase rather than at the transcriptional level under stress may underscore starch contents assayed. SnRK1 has a pivotal role in regulating carbohydrate metabolism and resource partitioning under stress^72,73^. In this study, *AtSnRK1.2* was up-regulated by osmotic and salinity stress after 6 hours of stress treatment. However, the transcript of *AtTPS1* and *AtSnRK1.1* did not change, indicating a possible delayed response to stress compared with *AtSnRK1.2*. The inconsistency in transcript changes of *AtSnRK1.1* and *AtSnRK1.2* might also be due to the specificity of these isoforms in terms of spatial expression and function^42,74^. In maize, salinity stress triggered more starch and sugar accumulation in both source and sink tissues and the transcripts of the *ZmTPSI.1.1* and *ZmTPSII.2.1* genes in the source leaf were down-regulated, while SnRK1 target genes *AKINβ* was affected mainly in the sink but not in the source.

## Conclusion

The present study observed profound changes in whole plant carbon use as an early response to short-term abiotic stress in *Arabidopsis*. Stress induces a reconfiguration of plant carbon fluxes primarily through starch in the source and growth substance in the sinks. We identified common and divergent patterns of carbon use triggered by cold, salinity and osmotic stress. We also emphasized the role of starch metabolism in stress response via enhancing sugar flexibility, together with sugar efflux in the source leaf. The stress-induced carbohydrates alteration in source was associated with transcriptional changes of key genes in the carbon deficit activated T6P/SnRK1 signaling pathway. How carbon reconfiguration is triggered by the T6P/SnRK1 or other stress-responsive signaling pathways deserve future studies.

## Methods

### Growth Conditions and Stress Treatment

*Arabidopsis thaliana* ecotype Colombia (Col-0) was grown hydroponically in Hoagland solution (12/12 h day/night, 21/23 °C). Five-week-old plants were transferred to Hoagland solution containing 100 mM NaCl, 200 mM NaCl, 150 mM mannitol, 300 mM mannitol, and 0 °C cold treatment for 5 hours before the ^14^CO_2_ labeling (n = 5).

### Starch content analysis

Source leaf or roots tissue from each plant was ground to a fine powder, and boiled three times successively in 1.5 mL 80% (v/v) ethanol for 10 min each. The starch content was measured following the protocol previously used^75^; briefly, The pellet was dried briefly, and was digested into glucose with alpha-amyloglucosidase and alpha-amylase (Roche Biosciences, Indianapolis, IN), then glucose content was measured at A340 nm absorbance and converted into starch content.

### ^14^CO_2_ Pulse-Chase Labeling

A single leaf ^14^CO_2_-labeling device for *Arabidopsis* was modified based on the rice device^17^ that was created following the prototype^28^. The ^14^CO_2_ feeding was carried out on the mature leaf of each twenty-leaf-old plant in the ‘leaf chamber’ in the middle of the photoperiod for 5 min. ^14^CO_2_ was generated from 0.08 MBq NaH^14^CO_3_ acidified with 200 μL of 10% (v/v) lactic acid in the reservoir chamber. The generated ^14^CO_2_ was pumped to a leaf chamber via the tubing system, where a single leaf of a plant was exposed to ^14^CO_2_. At the end of the feeding, 500 μL of 10% (v/v) KOH as used to stop ^14^CO_2_ generation and capture the residue ^14^CO_2_ in the chamber. The labeled leaf, unlabeled leaves and whole roots of each plant were harvested at 1 h, 7 h and 19 h after labeling.

### Fractionation of ^14^CO_2_-labelled plant tissue

Each sample was homogenized in liquid nitrogen, boiled for 10 min in successive 80%, 50%, and 20% (v/v) ethanol, and then separated into the soluble and insoluble fractions by centrifugation. The ethanol insoluble fractions (starch, protein, and cell wall) were digested with amylase and amyloglucosidase (10 U each per 200 μL of insoluble fraction, Roche Biosciences, Indianapolis, IN) to analyze for starch, and digested with pronase (10 U per 200 μl of insoluble fraction, *Streptomyces griseus*, Calbiochem®, Japan) to analyze for protein. The ethanol soluble solutions were fractionated into organic acids, amino acids, and sugars by ion-exchange chromatography using established methods in our lab. The ^14^C in each fraction was measured by liquid scintillation counting.

### RNA isolation and quantitative reverse transcription PCR

Five-week-old plants grown in Hoagland solution (12/12 h day/night, 21/23 °C) were transferred to Hoagland solution containing 200 mM NaCl, 300 mM mannitol for 0, 6, 12, and 24 h. Source leaf was harvested from each plant. Total RNA was extracted from each harvested sample with TRIzol® reagent (Invitrogen, Cansbad, CA). cDNA was synthesized using the high capacity cDNA Reverse Transcription Kit (Applied Biosystems, Vilnius, Lithuania). The primers for *Actin2* (housekeeping gene) amplification were forward, 5′-GGTGATGGTGTGTCT-3′; reverse, 5′-ACTGAGC ACAATGTTAC-3′. The primers for *SnRK1.1* were forward, 5′-CCGAATTGGGGATAGTCTGAAAATTGC-3′; reverse, 5′-CTCATCTACTCGTTTGAACATGAGAATTTAGCG-3′. The primers for *SnRK1.2* were forward, 5′-GAACTTCAGCTATACAAAGC -3′; reverse, 5′-GCGCATAGATCCAAGAAG-3′. The primers for *AtTPS1* were forward, 5′-GAGCTTAGAGAGAAGAGGAAGAGCAA-3′; reverse, 5′-TTCTAAACGCAAGTCATTCTCAGAGT-3′. The primers for *AtSWEET11* were forward, 5′-TCCTTCTCCTAACAACTTATATACCATG-3′, 5′-TCCTATAGAACGTTGGCACAGGA-3′. The primers for *AtAPL3* were forward, 5′-TCAGCACCATGCGATAGTAAAGC-3′; reverse, 5′-CAGTTGGTTTCTCAGAGAAATGGA-3′. Primers were optimized and the efficiency was calculated by making a standard curve with different dilutions of cDNA. One mL of each cDNA was used to amplify in 10 mL PCR reaction containing 2 × ABsolute^TM^ Blue qPCR SYBR Green ROX Mix (ABgene, foster city, CA) and 150 nM primers for genes mentioned above. The real-time PCR was performed in Applied Biosystems 7300 Real-time PCR System (Foster city, CA), the conditions were as follows: 10 min at 95 °C, 15 s at 95 °C, 40 cycles of 15 s at 95 °C, and 1 min at 60 °C. Source leaf from one plant represent one biological replicate. Three technical replicates were performed for each of the 3 biological replicates. ddCt method^76^ was used to analyze the expression of each gene based on the fold changes of relative transcripts in experimental sample compared with control sample.

### Statistical analysis

All tests for significant differences between treatment and control data were done using one-way ANOVA in the R environment. Heatmaps were generated using a web server Heatmapper (http://www.heatmapper.ca/expression/).

### Data availability

All of the materials, data and associated protocols will be made available upon request without preconditions. All data generated from this work, not presented in the figures is in the Supplemental Information file.

## Electronic supplementary material

Supplementary Information
