# Supplementary material for: A pivotal role for starch in the reconfiguration of 14C-partitioning and allocation in Arabidopsis thaliana under short-term abiotic stress
Source: Sci Rep. 2018 Jun 18;8:9314. doi: 10.1038/s41598-018-27610-y (PMC6006365; doi:10.1038/s41598-018-27610-y)
Supplement: Supplementary file 1 — Supplementary Information [file 41598_2018_27610_MOESM1_ESM.pdf]

Supplementary Information for

A pivotal role for starch in the reconfiguration of ^{14}C-partitioning and allocation in *Arabidopsis thaliana* under short-term abiotic stress

Shaoyun Dong, Joshua Zhang, Diane M. Beckles*

Department of Plant Sciences, University of California, One Shield Avenue, Davis, CA 95616, USA

*Corresponding Author:

Diane M Beckles,

Department of Plant Sciences,

University of California,

One Shields Avenue,

Davis, CA, 95616, USA.

E-mail: dmbeckles@ucdavis.edu

Supplementary Figures

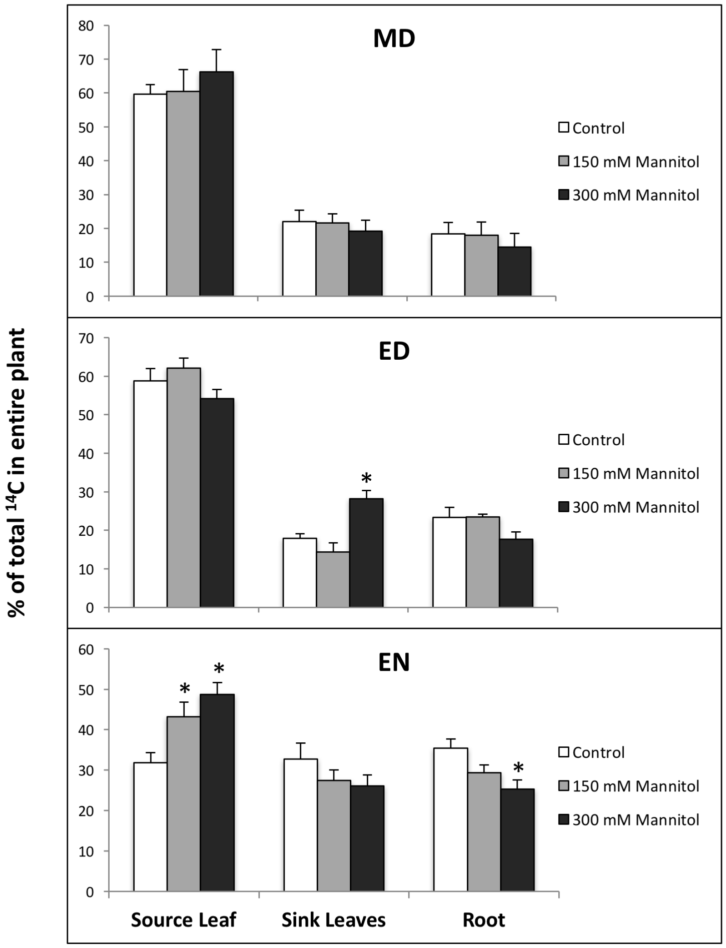

Supplementary Figure S1. The allocation of ^{14}C into different tissues of *Arabidopsis* grown under mild (150 mM) and severe (300 mM) Mannitol. The relative amount of ^{14}C exported from the labeled source leaf into unlabeled sink leaves and root tissues at the midday (MD), end of day (ED), and end of night (EN) were shown as a percentage of total labels in the entire plant. The asterisk indicates a significant difference between the control and mannitol-treated plants (n=5, $p<0.05$).

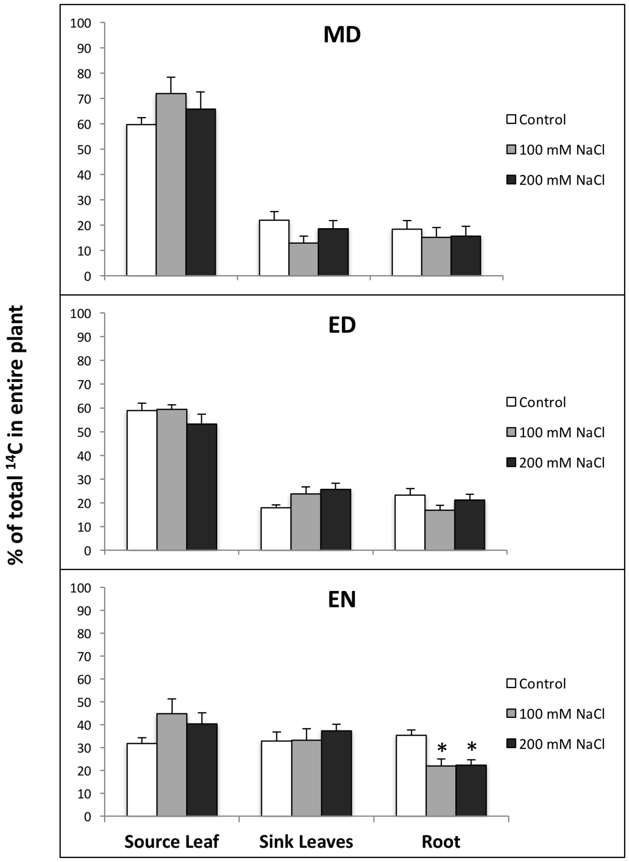

Supplementary Figure S2. The allocation of ^{14}C into different tissues of *Arabidopsis* grown under mild (100 mM) and severe (200 mM) NaCl. The relative amount of ^{14}C exported from the labeled source leaf into unlabeled sink leaves and root tissues at the midday (MD), end of day (ED), and end of night (EN) were shown as a percentage of total labels in the entire plant. The asterisk indicates a significant difference between the control and NaCl-treated plants (n=5, $p<0.05$).

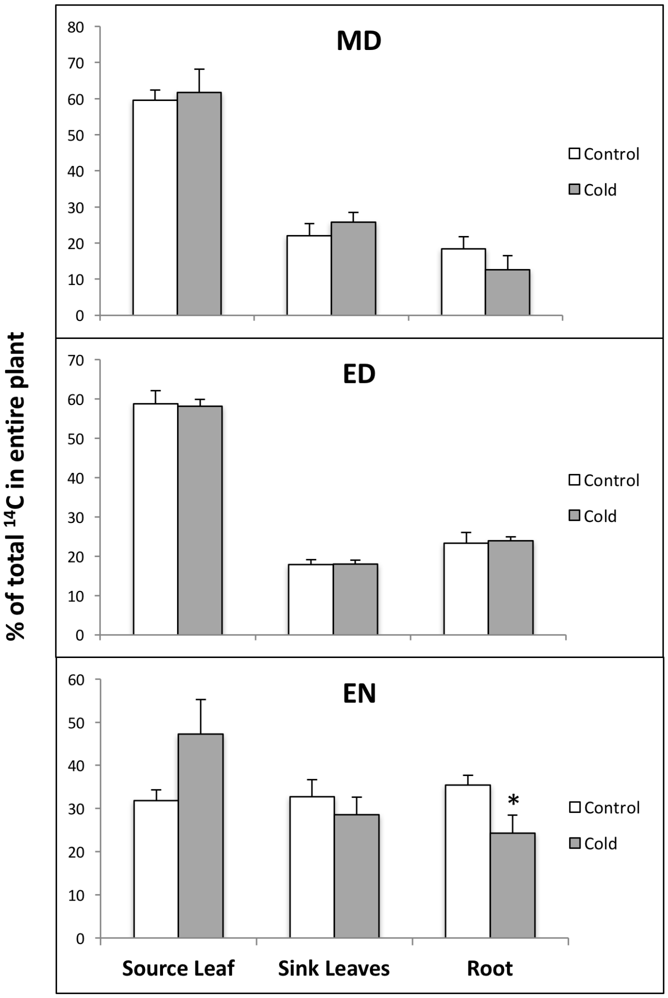

Supplementary Figure S3. The allocation of ^{14}C into different tissues of *Arabidopsis* grown under cold stress. The relative amount of ^{14}C exported from the labeled source leaf into unlabeled sink leaves and root tissues at the midday (MD), end of day (ED), and end of night (EN) were shown as a percentage of total labels in the entire plant. The asterisk indicates a significant difference between the control and cold-treated plants (n=5, $p<0.05$).

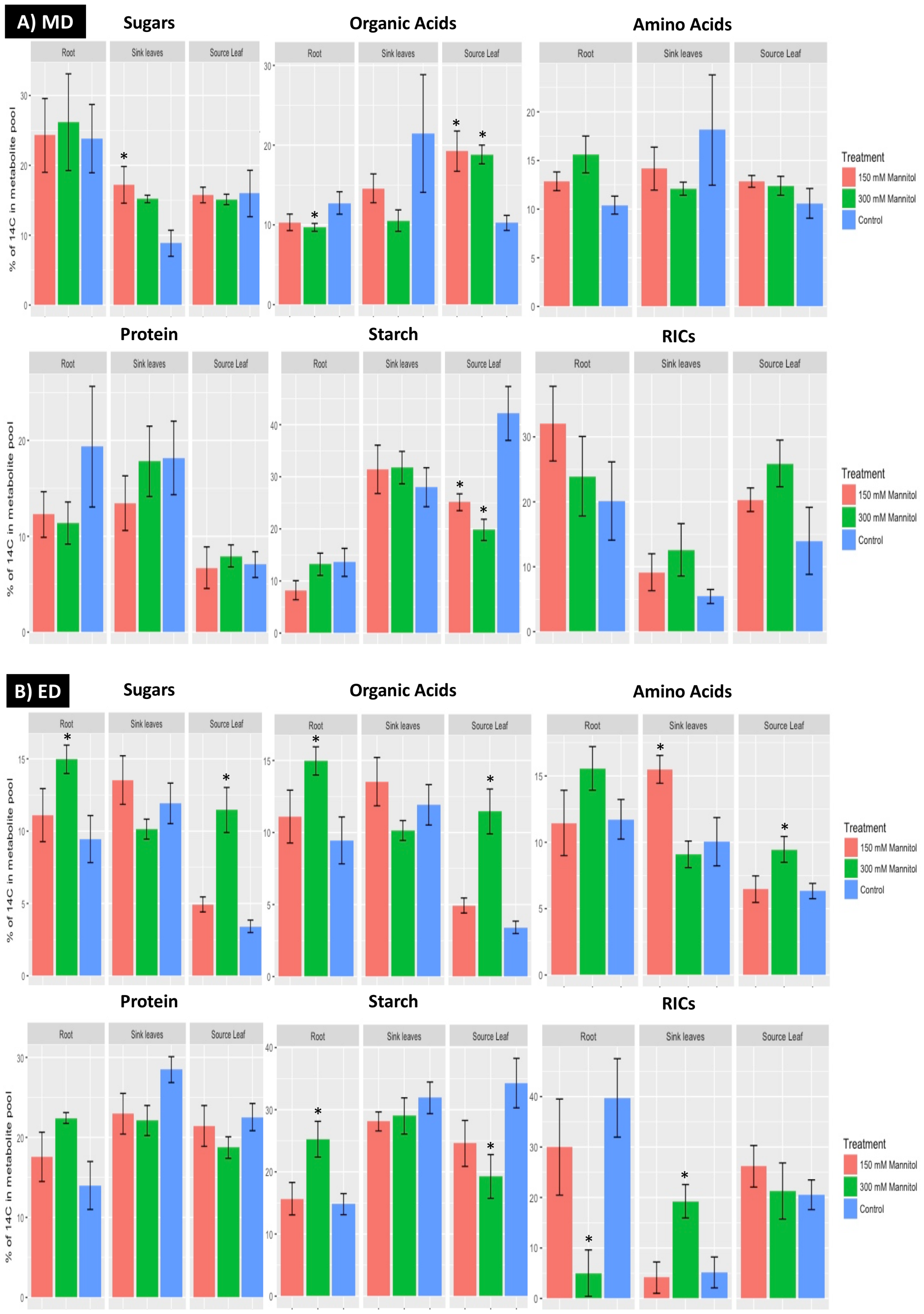
A) MD
Sugars
Organic Acids
Amino Acids
Protein
Starch
RICs
Root
Sink leaves
Source Leaf
% of 14C in metabolite pool
Treatment
150 mM Mannitol
300 mM Mannitol
Control
B) ED
Sugars
Organic Acids
Amino Acids
Protein
Starch
RICs

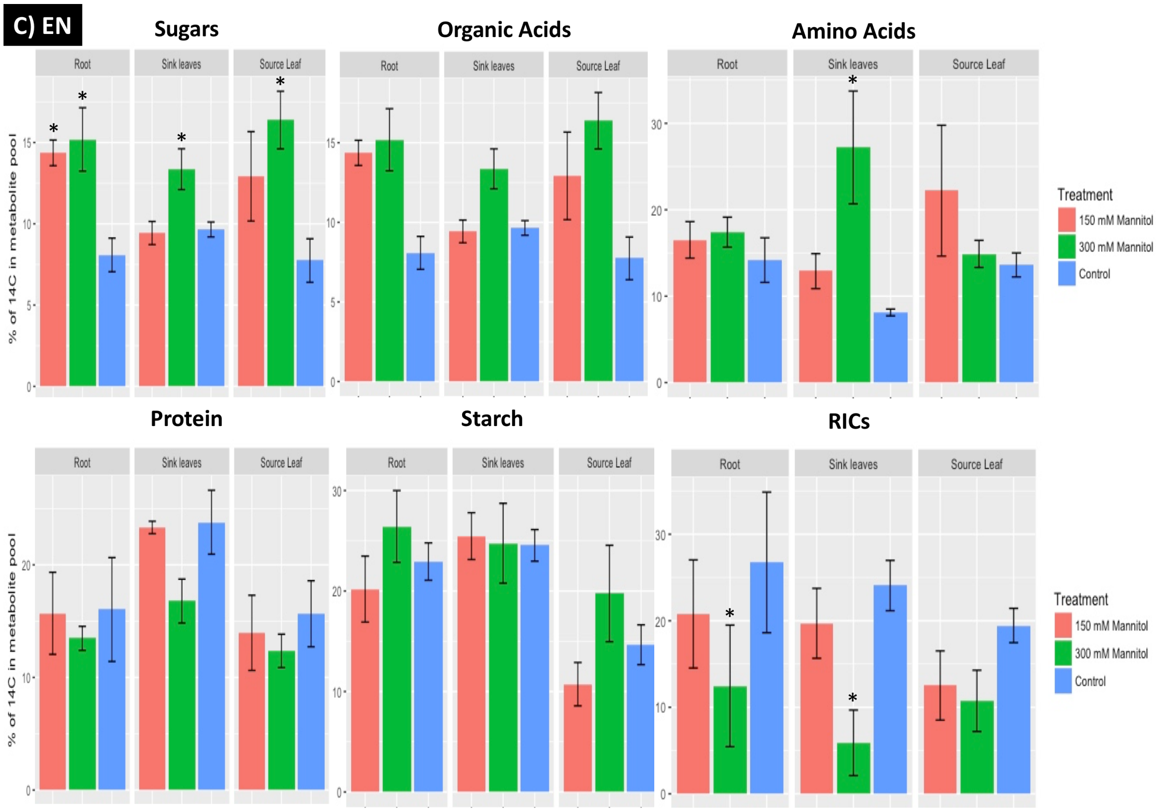

Supplementary Figure S4. ^{14}C partitioning in *Arabidopsis* under mild (150 mM) and severe (300 mM) mannitol stress. The incorporation of ^{14}C into sugars, starch, amino acids, protein, organic acids, and remaining insoluble compounds (RICs) in the source leaf, sink leaves, and root tissues at the A) midday (MD), B) end of day (ED), and C) end of night (EN) was determined. The total label in each tissue was set to 100%. The asterisks indicate a significant difference between the control and mannitol-treated plants (n=5, $P<0.05$).

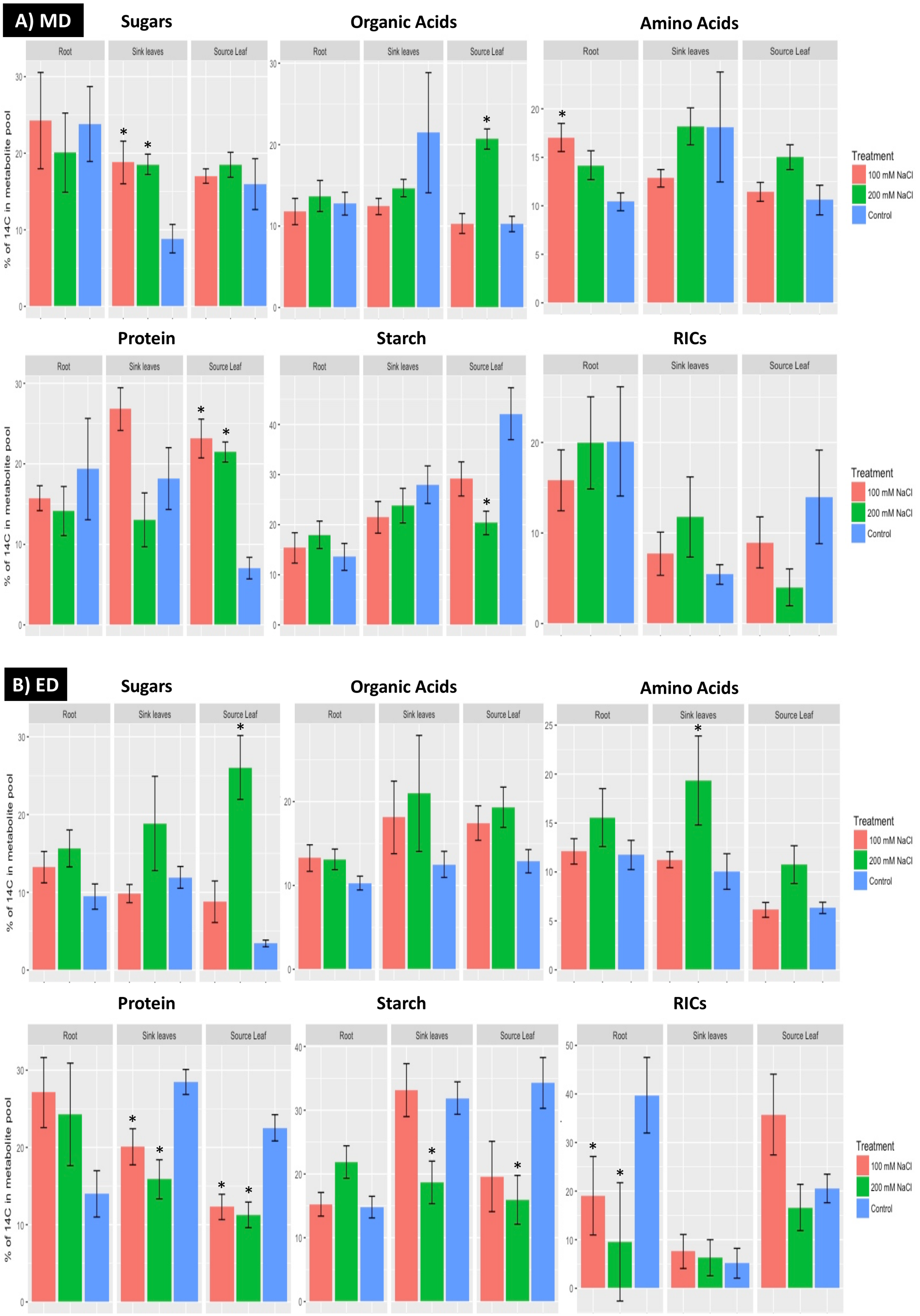

A) MD
Sugars
Organic Acids
Amino Acids
Protein
Starch
RICs
Root
Sink leaves
Source Leaf
% of 14C in metabolite pool
Treatment
100 mM NaCl
200 mM NaCl
Control
B) ED

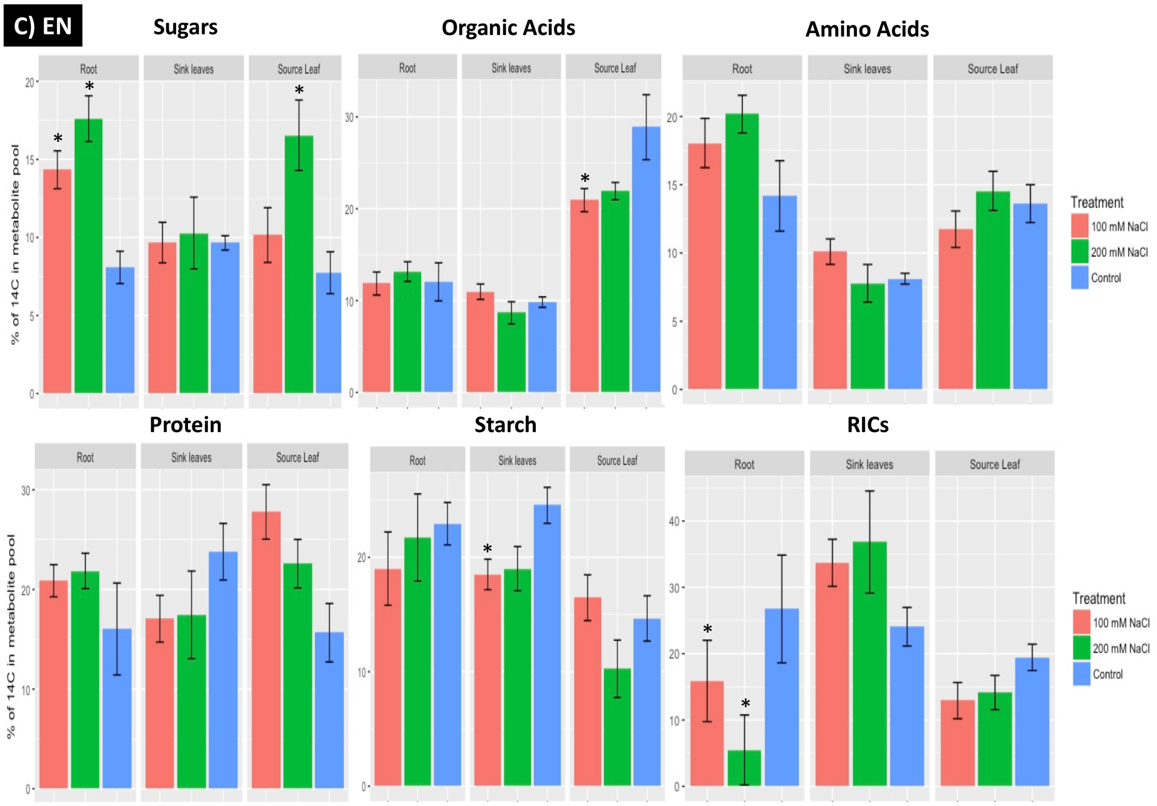

Supplementary Figure S5. ^{14}C partitioning in *Arabidopsis* under mild (100 mM) and severe (200 mM) NaCl. The incorporation of ^{14}C into sugars, starch, amino acids, protein, organic acids, and remaining insoluble compounds (RICs) in the source leaf, sink leaves, and root tissues at the A) midday (MD), B) end of day (ED), and C) end of night (EN) was determined. The total label in each tissue was set to 100%. The asterisks indicate a significant difference between the control and salinity-treated plants (n=5, $P<0.05$).

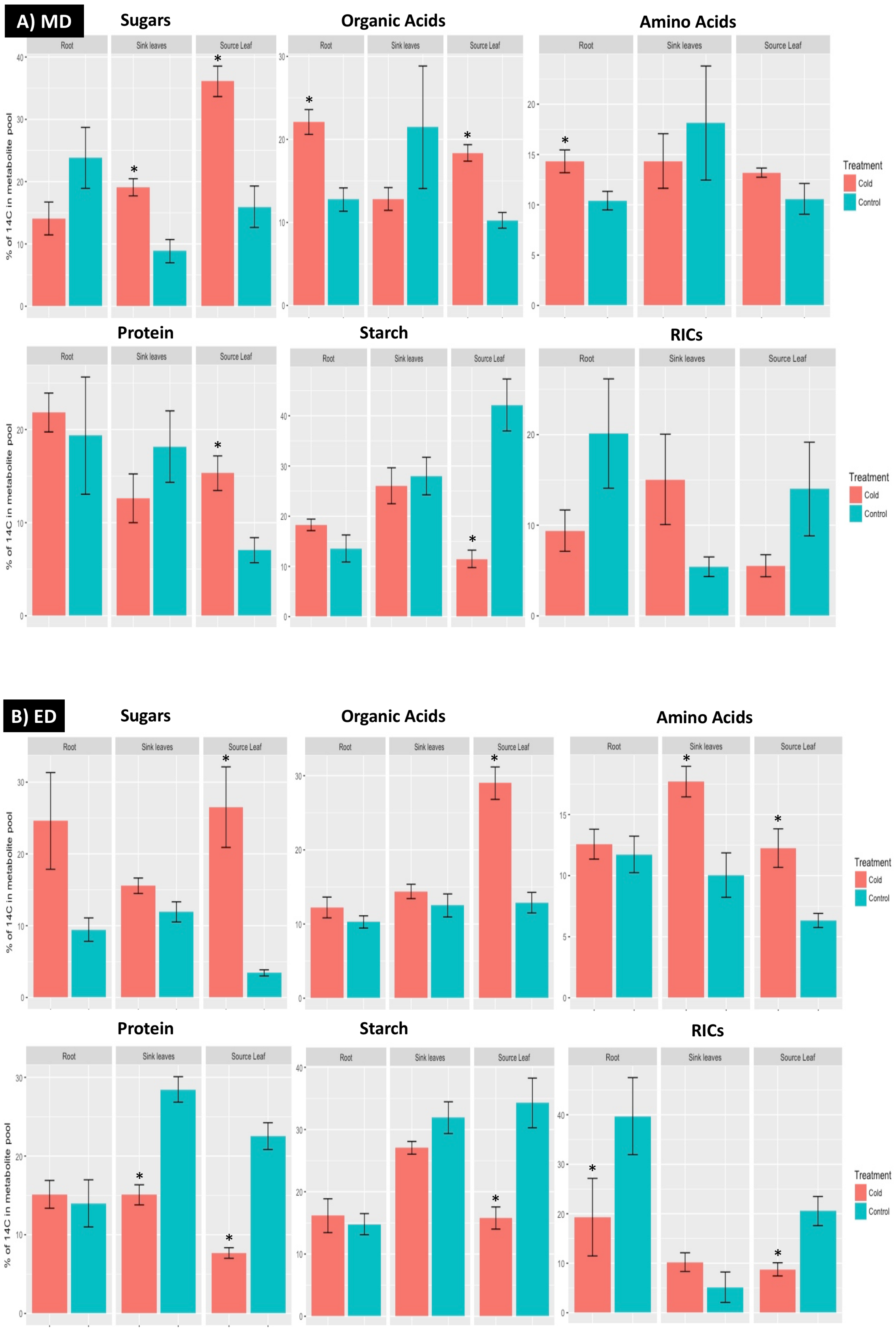
A) MD
Sugars
Organic Acids
Amino Acids
Protein
Starch
RICs
Root
Sink leaves
Source Leaf
% of 14C in metabolite pool
Treatment
Cold
Control
B) ED
Sugars
Organic Acids
Amino Acids
Protein
Starch
RICs
Root
Sink leaves
Source Leaf
% of 14C in metabolite pool
Treatment
Cold
Control

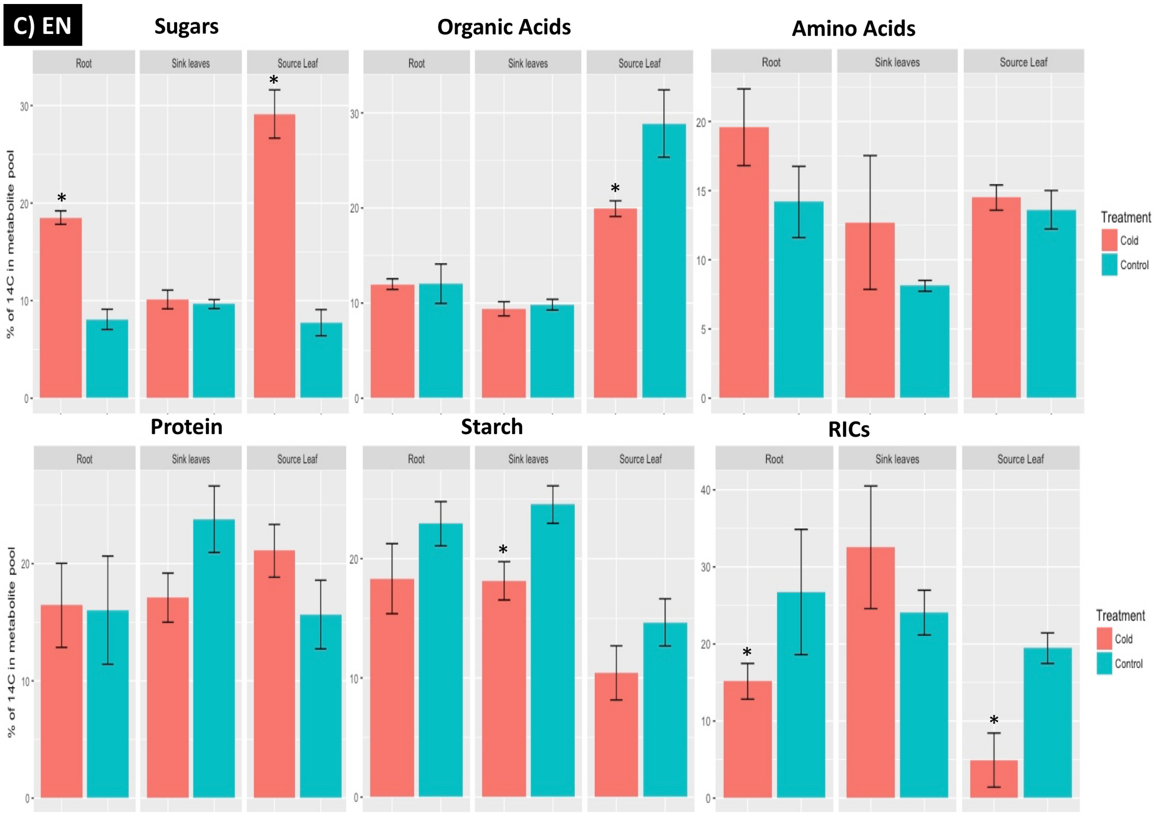

Supplementary Figure S6. ^{14}C partitioning in *Arabidopsis* under cold stress. The incorporation of ^{14}C into the sugars, starch, amino acids, protein, organic acids, and remaining insoluble compounds (RICs) in source leaf, sink leaves, and root tissues at the A) midday (MD), B) end of day (ED), and C) end of night (EN) was determined. The total label in each tissue was set to 100%. The asterisks indicate a significant difference between the control and cold-treated plants (n=5, $P<0.05$).

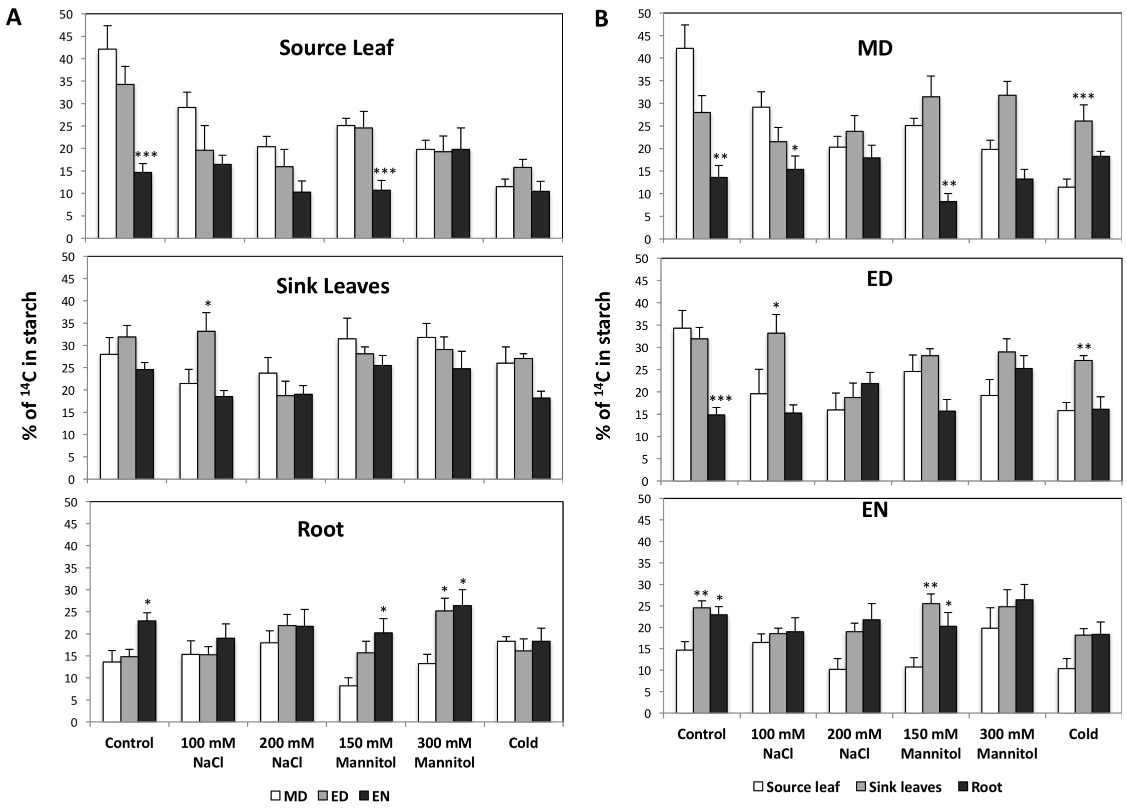

Supplementary Figure S7. Percentage of ^{14}C partitioned into starch under abiotic stress. A) The percentage of ^{14}C partitioning in starch over diurnal cycle. The asterisks indicate a significant difference between the MD and ED (or EN). **B) Differential of ^{14}C percentage partitioned into starch among tissues.** The asterisks indicate a significant difference between the LL and ULL (or Root) (n=5, '*', 0.01<P<0.05; '**', 0.001<P<0.01; '***', 0<P<0.001).
